# Cerebral tumor with hemi-dural enhancement as unique presentation of multiple myeloma: A case report

**DOI:** 10.1016/j.bas.2023.102730

**Published:** 2023-12-20

**Authors:** Rick H.G.J. van Lanen, Sandra M.H. Huijs, Alida A. Postma, Roel H.L. Haeren

**Affiliations:** a Department of Neurosurgery, Maastricht University Medical Centre+, Maastricht, the Netherlands; b School for Mental Health and Neuroscience (MHeNs), Maastricht University, Maastricht, the Netherlands; c Department of Neurology, Maastricht University Medical Centre+, Maastricht, the Netherlands; d Department of Radiology, Maastricht University Medical Centre+, Maastricht, the Netherlands

**Keywords:** Case report, Dura mater, Intracranial, Multiple myeloma, Plasmacytoma

## Abstract

**Introduction:**

Intracranial multiple myeloma (MM) is a rare manifestation of MM, a malignant plasma cell disorder that primarily affects bone marrow. Dural involvement in MM is even rarer and can manifest as a dural mass. We present a case of MM presenting as an intracranial dural tumor with primary hemi-dural involvement.

**Research question:**

This case report aims to investigate the clinical presentation, diagnostic challenges, and treatment approaches for intracranial multiple myeloma, with a focus on the extensive hemi-dural thickening and enhancement seen in this case.

**Material and methods:**

A 73-year-old male presented with progressive dysphasia and weakness. MRI revealed a solid left frontal mass with significant mass-effect. Hemi-dural thickening and enhancement was present along with invasion of the skull. The patient underwent surgical resection of the tumor with dural and bone reconstruction.

**Results:**

Histopathological examination confirmed MM diagnosis. Chemotherapy was started. Follow-up MRI showed complete tumor resection, but extensive hemi-dural thickening and enhancement persisted. Postoperative radiation therapy was considered.

**Discussion and conclusion:**

MM with primary dural involvement is rare and poses diagnostic challenges. Postoperative treatment involves chemotherapy, the role of surgery and radiotherapy is not established. The extensive hemi-dural thickening and enhancement observed in this case require further investigation, and a wait-and-scan policy was recommended instead of radiotherapy.

## Highlights

• Multiple myeloma as an intracranial tumor with primary dural involvement is rare.
• Diagnosis of multiple myeloma can be challenging as it may mimic common intracranial dural pathology.
• Hemi-cranial thickening and enhancement of the dura mater may be present.
• Wait-and-scan policy for hemi-dural pathology to limit radiation-induced complications.

## 1 Introduction

Intracranial multiple myeloma (MM) is a rare manifestation of multiple myeloma, a malignant plasma cell disorder that primarily affects bone marrow (Onodera et al., 2021). In rare cases, the disease can involve the central nervous system and result in the development of intracranial lesions. Intracranial MM can arise from the cranium, meninges or brain parenchyma (Azarpira et al., 2014). Dural involvement occurs when myeloma cells infiltrate the dura mater and form localized tumors. These tumors may arise from direct extension of adjacent bone lesions or as isolated dural lesions and can present as solitary masses or as multiple lesions spread throughout the dura (Cerase et al., 2008).

Understanding the characteristics and clinical implications of intracranial MM is crucial for accurate diagnosis and effective management. This condition poses significant challenges due to its rarity, varied presentation, and potential overlap with other intracranial tumors such as meningioma. This case report aims to investigate the clinical presentation, diagnostic challenges, and treatment approaches for a rare case of MM with primary dural involvement, with a focus on the extensive hemi-dural thickening and enhancement observed in the patient.

## 2 Case presentation

**Fig. 1:**
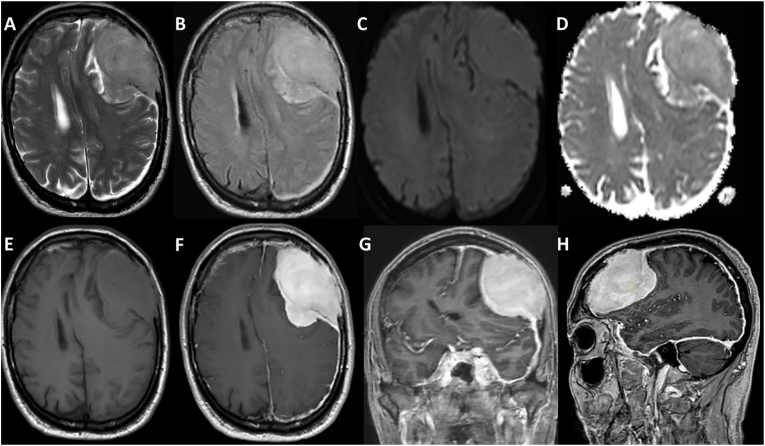
Magnetic Resonance Imaging (MRI) at presentation Axial T2-weighted (T2-w) (A), FLAIR (B), DWI (C), ADC (D), non-contrast axial T1-weighted (T1-w) (E) and axial, sagittal and coronal post-gadolinium T1-w images (F, G, H) are shown. A large extra-axial mass is shown with intermediate signal intensity on T2-w. No diffusion restriction is present. Only mild edema is present. There is significant mass effect with subfalcine herniation. There is osseous and focal dural involvement, with striking thickened and enhancing dura along the left hemispheric convexity. Note that the dura is located within the tumor and thus detached from the skull by the tumor.

A 73-year-old male without relevant history presented with slowly progressive expressive dysphasia, bradyphrenia and mild right-sided weakness for six weeks. On physical examination, the patient had an expressive dysphasia and mild right-sided weakness. Magnetic resonance imaging (MRI) of the brain (Fig. 1) showed a large, T2 hyperintense solid extra-axial left frontal mass with inward displacement of the frontal cortex and brain. Following gadolinium administration relative homogeneous contrast-enhancement of the lesion was present with striking enhancement and thickening of the left hemispheric dura with local inward displacement of the left-sided dura and extension of the tumor in the dura and the skull. No restrictive diffusion was present. Work-up with Computed Tomography (CT) scans of the thorax and abdomen showed no primary tumor, but some faint heterogeneity of the lumbar skeleton. A differential diagnosis of meningioma, lymphoma, solitary fibrous tumor, and dural/osseous metastasis was established.

**Fig. 2:**
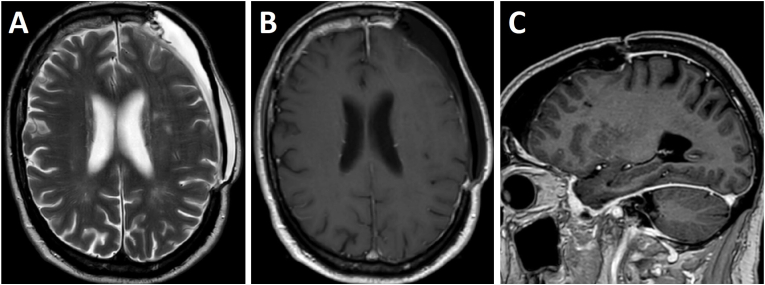
Postoperative Magnetic Resonance Imaging (MRI) at three months. Axial T2 (A) and T1-contrast-enhanced (B) MRI showing complete removal of the tumor with diminished left-sided dural thickening and enhancement extending to the basal dura mater (sagittal view in C).

The patient underwent surgical resection of the tumor via a left frontal craniotomy with removal of the macroscopically affected dura and bone. Due to the invasive nature of the extradural tumor, which caused extensive erosion in the skull, including the tabula externa, a polymethyl-methacrylate (PMMA) based cranioplasty was performed to replace the cranial flap. Initially, a PMMA cast was molded over the intact skull area intended for the craniotomy and tumor resection following the opening of the skin and temporal muscle. After the tumor resection, this cast was used as a negative mold to reconstruct the resected skull bone, resulting in a positive restoration. The deepest part of the intradural tumor was slightly invasive into the brain, yet was resected completely. Direct post-operative MRI showed complete removal of the tumor with persisting profound left-sided hemi-dural contrast-enhancement. Histopathological examination showed a cellular tumor composed of plasma cells proliferation, expressing monoclonal kappa light chain, but not lambda light chain, indicative of neoplastic plasma cells, consistent with plasmacytoma. A systematic work-up led to the diagnosis of multiple myeloma (MM). The hematologist started with daratumumab-lenalidomide-dexamethasone therapy. The patient completely recovered from his neurological symptoms. A wait-and-scan policy was established for the hemi-dural abnormalities. At three months follow-up, neurological symptoms were absent, MRI showed postoperative changes and no recurrence of the intracranial lesion. Notably, the left-sided dural enhancement and thickening was diminished (Fig. 2).

## 3 Discussion

MM presenting as an intracranial tumor is very rare, especially with primary dural involvement (Méndez et al., 2010). A comprehensive literature search was used, based on the search terms ‘multiple myeloma’, ‘plasmacytoma’, ‘intracranial’, ‘skull’, ‘calvarium’, ‘dura’ and ‘dura mater’ and performed using the Preferred Reporting Items for Systematic reviews and Meta-Analyses (PRISMA) guidelines (Page et al., 2021). Although there are several reports of multiple myeloma presenting as an intracranial mass, only a limited number of studies have specifically reported on cases with primary dural involvement (Azarpira et al., 2014; Cerase et al., 2008; Gallina et al., 2004; Haegelen et al., 2006; Hasturk et al., 2013; Lasocki et al., 2015; Méndez et al., 2010; Nakai et al., 1999; Onodera et al., 2021; Rahmah et al., 2009; Sahin et al., 2006; Terada, 2009; Tsang et al., 2006; Wavre et al., 2007). Interestingly, no literature was found specifically addressing the hemi-dural enhancement observed in our case.

Diagnosis of intracranial MM with primary dural involvement may be difficult as they mimic common intracranial dural lesions such as meningiomas and lymphoma (Lasocki et al., 2015). There is no pathognomonic radiological feature of MM on CT or MRI. Features that may suggest MM are a bulky soft tissue mass with iso-to hyperintense T1-and iso-to hypointense T2-weighted MRI signal, along with restricted diffusion due to high cellularity (Cerase et al., 2008). On CT-scan, sharp tumor margins, lack of bony sclerosis and paucity of periosteal reaction is suggestive of MM or plasmacytoma. Although the “dural tail” sign has been considered as a highly specific feature of meningioma, MM can show the same findings on MRI (Nakai et al., 1999). CT and MR imaging are complementary to each other in order to assess both bony and soft tissue involvement (Azarpira et al., 2014; Cerase et al., 2008). As these tumors are likely to invade and destruct the bone, reconstruction is required, as described in this case. The remarkable and extensive dural enhancement observed in our case has not been previously reported. This finding may indicate the presence of reactive tissue or potentially indicate a more widespread disease process.

An intracranial brain lesion can be the first manifestation of MM, or intracranial plasmacytoma may gradually transform into MM (Terada, 2009). Increased intracranial pressure or focal neurological deficit is the usual clinical presentation (Wavre et al., 2007). Definite diagnosis requires histopathological examination (Lasocki et al., 2015). If the disease is found to progress to multiple myeloma, the patients should be staged and treated with treatment protocols appropriate for multiple myeloma. While local recurrence is observed in 12% of the patients within 10 years, a second lesion is detected in 15%. Systemic MM develops at a rate of 44–69% within 3 years (Hasturk et al., 2013). Chemotherapy is described as the preferred treatment strategy, whereas the role of surgical resection and radiotherapy has not been established (Lasocki et al., 2015; Terada, 2009). Currently, there is a lack of literature regarding the treatment approach for the diffuse hemi-dural enhancement and thickening observed in our case. The case was discussed in the neuro-oncology multidisciplinary team, where radiotherapy was considered for the dural enhancement/thickening. However, radiotherapy was discarded as it would require hemi-cranial radiation. Such a large radiation target-volume has associated drawbacks and risks, such as fatigue, leukoencephalopathy, neurocognitive and functional decline, impairing quality of life (Wujanto et al., 2021). This has to be weighed against the possibility of tumor recurrence. The diminished dural enhancement/thickening at three-month follow-up is promising and may suggest effectiveness of current therapy or regression of reactive enhancement.

Our report has limitations. First of all, owing to the fact this article is a case report, this study is lacking generalizability, has limited ability to establish a cause-effect relationship between treatment and effects the follow-up the duration is confined. Moreover, there is a, lack of literature to guide treatment decision-making very a rare entity such as we presented here. However, this report may serve as an example for similar cases and prompt additional publications on the diagnostic challenges of intracranial multiple myeloma and treatment considerations for hemi-dural enhancement.

## 4 Conclusion

Multiple myeloma presenting as an intracranial tumor is very rare, especially with primary dural involvement. Diagnosis may be difficult as they mimic common intracranial dural lesions such as meningiomas and lymphoma. There is currently no established treatment for intracranial MM. At present, tailored combined therapy with chemotherapy, radiotherapy, and surgical resection is chosen as appropriate for each patient. Hemi-dural thickening and enhancement can be present and may suggest reactive tissue or more widespread disease. We suggest a wait-and-scan policy for such hemi-dural pathology opposed to hemi-cranial radiotherapy.

## Funding, grant/award

None.

## Availability of data and material

Data are available upon reasonable request.

## Code availability

Not applicable.

## Ethical approval statement

Not applicable; the article reports the description of an exceptional case, where no research, no intervention, or procedure away from clinical practice has been performed on the subject. Thus, no ethics approval has been applied, according to the institution's policy.

## Consent for publication

Written informed consent was obtained from the patient for publication of this case report and any accompanying images. A copy of written consent is available for review by the Editor of the journal if necessary.

## Author contributions

R.L. contributed to conception and design of the study, acquisition and analysis of data, drafting the manuscript and figure. S.H. contributed to conception and design of the article. A.P. contributed to contributed to conception and design of the study, drafting the manuscript and figure. R.H. contributed to conception and design of the study, acquisition and analysis of data, drafting the manuscript and figure.

## Declaration of competing interest

The authors declare that they have no known competing financial interests or personal relationships that could have appeared to influence the work reported in this paper.
