# Supplementary material for: Cerebral tumor with hemi-dural enhancement as unique presentation of multiple myeloma: A case report
Source: Brain Spine. 2023 Dec 20;4:102730. doi: 10.1016/j.bas.2023.102730 (PMC10951707; doi:10.1016/j.bas.2023.102730)
Supplement: Multimedia component 1 [file mmc1.docx]

**Literature review**

**Research protocol**

Our review protocol consisted of a search strategy, screening criteria for titles and abstracts, and screening criteria for full-text articles. The search strategy was based on the search terms ‘multiple myeloma’, ‘plasmacytoma’, ‘intracranial’, ‘skull’, ‘calvarium’, ‘dura’ and ‘dura mater’ and performed using the Preferred Reporting Items for Systematic reviews and Meta-Analyses (PRISMA) guidelines. Search databases were Medline (using PubMed), Embase (using Ovid), the Cochrane Library and clinicaltrials.gov. Search queries were optimized for each specific database. After deleting duplicate records, titles and abstracts were screened and included if they represented studies of patients with intracranial multiple myeloma with primary dural involvement. Exclusion criteria were no studies with patients, no full text available, no English full-text available, no primary cranial or dural involvement, and no structural imaging. Of the remaining records, full-text articles were assessed according to the same criteria. The first author (RvL) set up the research protocol and performed the search.

**Eligibility criteria, search strategy and article selection**

Study selection was based on the inclusion criteria. Publication dates were limited to the last 25 years. We applied no additional restrictions to the search. Primary data source was Medline, using PubMed. We aimed at maximum sensitivity and kept search terms as broad as possible by also adding them as “free terms” alongside “MeSH terms”. Full search strategies are detailed for each database as follows. The Medline query was: “("multiple myeloma"[MeSH Terms] OR "multiple myeloma"[All Fields]) OR “("plasmacytoma"[MeSH Terms] OR " plasmacytoma"[All Fields]) AND ("intracranial"[All Fields] OR "skull"[All Fields] OR "calvarium"[All Fields]) AND ("dura"[All Fields] OR "dura mater"[All Fields])”. Adding the search term “Humans” as either a [MESH Terms] or [All Fields] resulted in the exclusion of relevant articles, therefore we did not use this search term, making it necessary to exclude studies without included cases manually. The Ovid thesaurus terms were: (“multiple myeloma” OR “plasmacytoma”) AND (“intracranial” OR “skull” OR “calvarium”) AND (“dura” OR “dura mater”). The Cochrane Library keywords were: (multiple myeloma OR plasmacytoma):ti,ab,kw AND (intracranial OR skull OR calvarium):ti,ab,kw AND (dura OR dura mater): ti,ab,kw. The ClinicalTrials.gov query consisted of free text: (multiple myeloma OR plasmacytoma) AND (intracranial OR skull OR calvarium) AND ( dura OR dura mater). The last search was last performed on June 12, 2023.


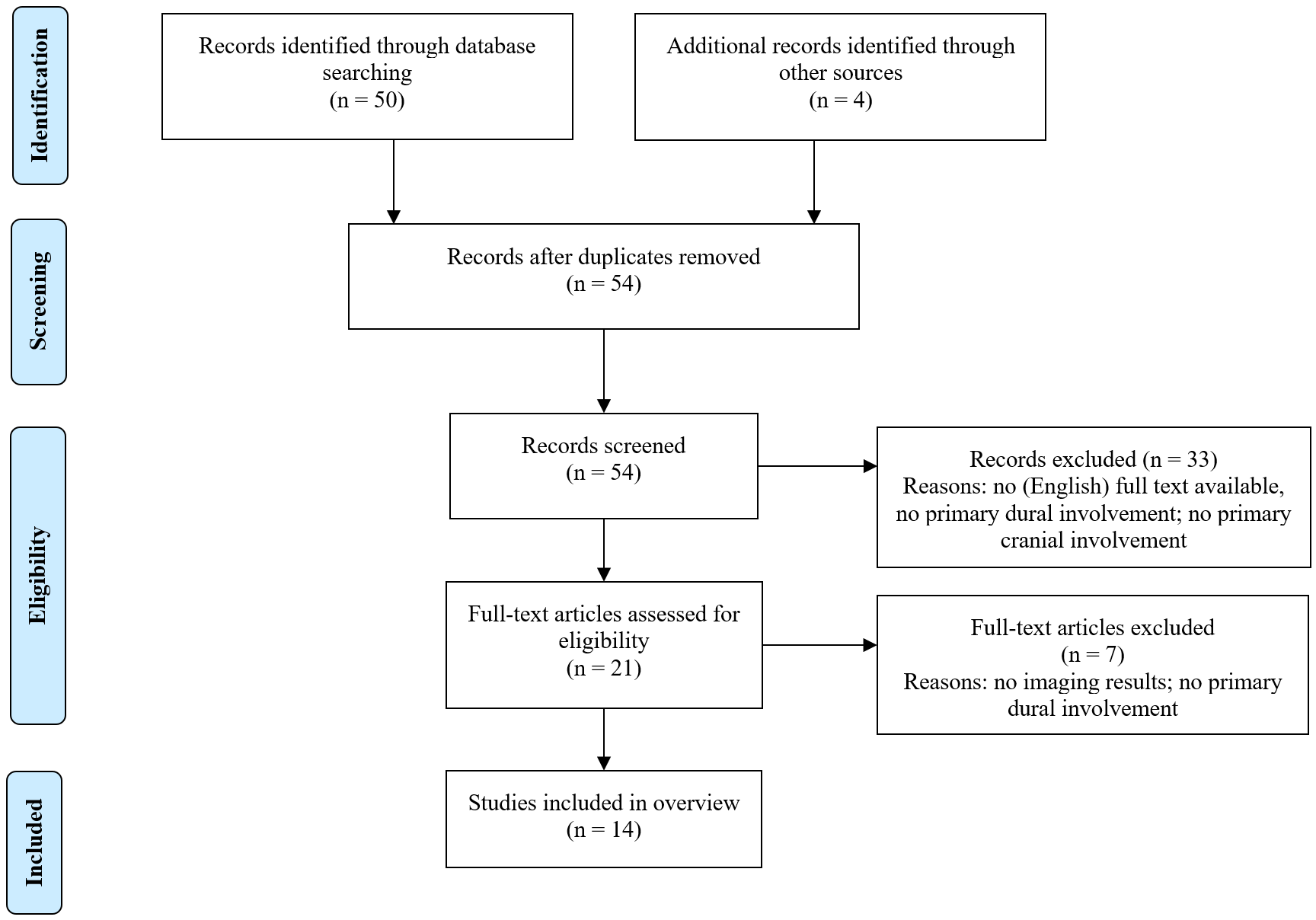


Figure 1. Flow diagram of the article search, adapted after the PRISMA guidelines.

Studies included in overview: (Azarpira et al., 2014; Cerase et al., 2008; Gallina et al., 2004; Haegelen et al., 2006; Hasturk et al., 2013; Lasocki et al., 2015; Méndez et al., 2010; Nakai et al., 1999; Onodera et al., 2021; Rahmah et al., 2009; Sahin et al., 2006; Terada, 2009; Tsang et al., 2006; Wavre et al., 2007)

**References**

Azarpira, N., Noshadi, P., Pakbaz, S., Torabineghad, S., Rakei, M., & Safai, A. (2014). Dural plasmacytoma mimicking meningioma. *Turkish Neurosurgery*, *24*(3), 403–405. https://doi.org/10.5137/1019-5149.JTN.5735-12.1

Cerase, A., Tarantino, A., Gozzetti, A., Muccio, C. F., Gennari, P., Monti, L., Di Blasi, A., & Venturi, C. (2008). Intracranial involvement in plasmacytomas and multiple myeloma: a pictorial essay. *Neuroradiology*, *50*(8), 665–674. https://doi.org/10.1007/s00234-008-0390-x

Gallina, P., Mascalchi, M., Mouchaty, H., Buccoliero, A., & Perrini, P. (2004). Misleading imaging features of intracranial dural plasmacytoma: report of two cases. *British Journal of Neurosurgery*, *18*(6), 643–646. https://doi.org/10.1080/02688690400022748

Haegelen, C., Riffaud, L., Bernard, M., Carsin-Nicol, B., & Morandi, X. (2006). Dural plasmacytoma revealing multiple myeloma. *Journal of Neurosurgery*, *104*(4), 608–610. https://doi.org/10.3171/jns.2006.104.4.608

Hasturk, A. E., Basmaci, M., Erten, F., Cesur, N., Yilmaz, E. R., & Kertmen, H. (2013). Solitary Dural Plasmacytoma Mimicking Meningioma and Invading Calvarium. *Journal of Craniofacial Surgery*, *24*(2), e175–e177. https://doi.org/10.1097/SCS.0b013e31827c85ba

Lasocki, A., Gangatharan, S., Gaillard, F., & Harrison, S. J. (2015). Intracranial involvement by multiple myeloma. *Clinical Radiology*, *70*(8), 890–897. https://doi.org/10.1016/j.crad.2015.03.014

Méndez, C. E., Hwang, B. J., Destian, S., Mazumder, A., Jagannath, S., & Vesole, D. H. (2010). Intracranial Multifocal Dural Involvement in Multiple Myeloma: Case Report and Review of the Literature. *Clinical Lymphoma Myeloma and Leukemia*, *10*(3), 220–223. https://doi.org/10.3816/CLML.2010.n.035

Nakai, Y., Yanaka, K., Iguchi, M., Fujita, K., Narushima, K., Meguro, K., Doi, M., & Nose, T. (1999). A case of multiple myeloma presenting with a subcutaneous mass: significance of “dural tail sign” in the differential diagnosis of the meningeal tumors. *No Shinkei Geka. Neurological Surgery*, *27*(1), 67–71.

Onodera, K., Kurisu, K., Takebayashi, S., Sakurai, J., Kobayashi, T., Kobayashi, R., Goto, S., & Takizawa, K. (2021). Intracranial plasmacytoma arising from dura mater secondary to multiple myeloma and presenting with sudden lethal intracerebral hemorrhage: A case report and literature review. *Surgical Neurology International*, *12*, 55. https://doi.org/10.25259/SNI_693_2020

Rahmah, N., Brotoarianto, H., Andar, E., Kusnarto, G., Muttaqin, Z., & Hongo, K. (2009). Dural plasmacytoma mimicking meningioma in a young patient with multiple myeloma. *Biomedical Imaging and Intervention Journal*, *5*(2). https://doi.org/10.2349/biij.5.2.e5

Sahin, F., Saydam, G., Ertan, Y., Calli, C., Dönmez, A., & Tombuloglu, M. (2006). Dural plasmacytoma mimicking meningioma in a patient with multiple myeloma. *Journal of Clinical Neuroscience*, *13*(2), 259–261. https://doi.org/10.1016/j.jocn.2005.03.033

Terada, T. (2009). Multiple myeloma presenting as an intracranial plasmacytoma: a case report. *Cases Journal*, *30*(2), 9110. https://doi.org/10.1186/1757-1626-2-9110

Tsang, C.-S., Ho, L. C., & Tan, T.-C. (2006). Intracranial multiple myeloma involving the dura. *Journal of Clinical Neuroscience*, *13*(1), 122–123. https://doi.org/10.1016/j.jocn.2005.02.008

Wavre, A., Baur, A. S., Betz, M., Mühlematter, D., Jotterand, M., Zaman, K., & Ketterer, N. (2007). Case study of intracerebral plasmacytoma as an initial presentation of multiple myeloma. *Neuro-Oncology*, *9*(3), 370–372. https://doi.org/10.1215/15228517-2007-008
